# Unveiling Neurobrucellosis: A Case Report Emphasizing Early Diagnosis for Better Outcomes

**DOI:** 10.1099/acmi.0.000705.v3

**Published:** 2024-02-13

**Authors:** Sivakumar Haripriya, Shikhir Malhotra, Ravisekhar Gadepalli, Vibhor Tak, Bharat Kumar

**Affiliations:** ^1^​ Department of Microbiology, All India Institute of Medical Sciences (AIIMS), Jodhpur, Rajasthan 342005, India; ^2^​ Department of General Medicine, All India Institute of Medical Sciences (AIIMS), Jodhpur, Rajasthan 342005, India

**Keywords:** *Brucella melitensis*, *Brucella *serology, neurobrucellosis, zoonosis

## Abstract

**Introduction.:**

Brucellosis is a pervasive zoonotic disease causing considerable human morbidity worldwide. This report focuses on a case of neurobrucellosis in a rural Indian patient, emphasizing the need for timely microbiological confirmation given its nonspecific clinical presentation.

**Case Presentation.:**

A 55-year-old rural Indian farmer presented with a 3 week history of insidious, low-grade fever, myalgia, and arthralgia. He developed acute right-sided weakness and neurological symptoms, including disorientation and neck rigidity. Laboratory tests indicated abnormal blood counts, elevated inflammatory markers, and liver dysfunction. Cerebrospinal fluid analysis showed pleocytosis with lymphomononuclear cells and elevated protein levels. Blood cultures eventually grew Gram-negative coccobacilli. Serological tests confirmed neurobrucellosis. Prompt antibiotic therapy led to clinical and laboratory improvement.

**Conclusion.:**

This case underscores the importance of recognizing neurobrucellosis, particularly in endemic areas, given its nonspecific clinical presentation. Early microbiological diagnosis, supported by positive blood cultures and serological tests, was crucial. The patient’s rapid response to appropriate antibiotics emphasizes the significance of timely recognition and management.

## Data Summary

No new data, tools, software or code was used for this case report. Supplementary data file has reports of VITEK MS (bioMérieux Inc) (data: S1.a, available in the online version of this article) and VITEK 2.0 Compact (bioMérieux Inc) (data: S1.b) by which the identification of the isolated organism (*Brucella melitensis*) was performed. The serology for both anti-Brucella IgM and IgG antibody (Calbiotech, Inc Brucella IgM and IgG ELISA Kit) (data: S1.c) were performed.

## Introduction

Brucellosis is the most frequent bacterial zoonosis, causing around 500 000 human illnesses each year [1]. The disease has a wide global distribution and is considered a regionally emerging zoonotic disease. It is included on the World Health Organization’s list of neglected tropical zoonotic illnesses [2]. *Brucella* is a tiny aerobic intracellular coccobacillus that causes abortion and infertility in the host animal’s urogenital tract. Human brucellosis, a zoonotic disease mainly caused by four species *Brucella melitensis* (from sheep)*, B. abortus* (from cattle)*, B. suis* (from pig), and *B. canis* (from dog) [3]. Human brucellosis is transmitted by ingestion of contaminated milk, cheese and dairy products, by aerosol and occupational exposure to infected sheep, cattle and dogs [4]. Human brucellosis is a multisystem illness with a wide range of clinical symptoms and consequences. There are three types of brucellosis: acute (septicemia), subacute (secondary localisation), and chronic (>1 year) [5]. Neurobrucellosis may occur at any stage of the disease and may lead to encephalitis, meningoencephalitis, myelitis, cerebral venous thrombosis, and psychiatric symptoms [4]. Thus, a timely microbiological confirmed diagnosis is of utmost importance. As a result, we present a case of a 55-year-old male with neurobrucellosis and review of the relevant literature.

## Presentation of case

A 55-year-old male resident of Pipar city (rural countryside), Jodhpur, India, farmer by occupation presented to our tertiary healthcare centre with the symptoms of fever of 3 weeks duration, which was insidious in onset, persistent, low grade and relieved on taking antipyretics. Fever was accompanied by drowsiness, lethargy, myalgia, arthralgia (large joints) for the last 5 days of the illness before presenting to the healthcare facility. It was not associated with headache, projectile vomiting, visual disturbance, seizure, loss of consciousness or abnormal body movements. The patient received antibiotics for the aforementioned complaints by a local practitioner. However, the prescription of which could not be traced and no clinical improvement was noted. Patient reported consuming unpasteurized goat milk, handling and residing in close proximity to the livestock. But had no history of contact with tuberculosis patients.

On the fourth day of admission, the patient developed an acute onset of weakness on the right side of the body, which was more prominent in the upper limb as compared to the lower limb. The patient was hemodynamically stable with a body temperature of 38.7 °C, but was disoriented to time, place and person. Physical examination of the patient revealed pallor, icterus and neck rigidity. Gradual weight loss of around 10–15 kgs was noted in the past 1 year. Motor examination revealed, hypotonia in upper and lower limbs, grade II power in muscles of all limbs with sluggish deep tendon reflexes. Sensory examination was normal. No other neurological complaints were noted.

Laboratory parameters revealed an elevated neutrophil count of 11882 cells mm^−3^ (84.8 %), reduced lymphocyte 1680 cells mm^−3^ (12 %) count and thrombocytopenia 78560 cells mm^−3^ was noted. Inflammatory markers such as C-reactive protein and erythrocyte sedimentation rate were found to be elevated, i.e. 120.1 mg l^−1^ and 27 mm per hour respectively. Liver function test revealed an elevated aspartate transaminase: 145.4 IU l^−1^, alanine transaminase: 83.6 IU l^−1^, alkaline phosphatase: 374 IU l^−1^, total bilirubin: 4.40 mg dl^−1^, direct bilirubin: 2.65 mg dl^−1^ and indirect bilirubin: 1.75 mg dl^−1^ with a blood ammonia level of 84 µg dl^−1^. Renal function tests were within normal limits.

In view of the persistent deteriorating neurological symptoms, patient underwent lumbar puncture and the cerebrospinal fluid examination (CSF) of the patient depicted pleocytosis (12 cells mm^−3^) with lymphomononuclear (99 %) predominance, elevated protein (174 mg dl^−1^) and reduced glucose (22 mg dl^−1^) levels, but in contrast to this the CSF culture was negative for bacterial and fungal growth.

With pyrexia of unknown origin (PUO) as the working diagnosis, human immunodeficiency virus 1 and 2 antibody, japanese encephalitis IgM serology, scrub typhus IgM serology, non-structural one dengue antigen, typhoid WIDAL test and pan plasmodium lactate dehydrogenase and histidine rich protein-2 malarial antigens were found to be negative. Since the patient had deranged liver function test, Hepatitis A, B, C, E serology testing were done and were found to be non-reactive. In the tuberculosis workup, CSF was negative for Acid Fast Bacilli (AFB) and Cartridge-Based Nucleic Acid Amplification Test (CBNAAT). Interestingly, the radiological findings did not reveal any significant abnormality.

As a routine diagnostic workup for PUO, paired blood culture bottles were sent to the department of Microbiology on three consecutive days. Time to positivity for first set was around 02 days 21 h, second set around 03 days 16 h and third set 03 days 21 h post-incubation respectively. All the samples were processed inside Biosafety cabinet 2b with standard respiratory precautions: N95 mask, lab coat, gloves. Initial Gram-staining of all three pairs of the blood culture bottles demonstrated Gram-negative coccobacilli under 1000× magnification. Subculture from the positive blood culture bottles were done on to 5 % sheep blood agar and chocolate agar. The plates were incubated in CO_2_ and routine aerobic incubator for 24 and 48 h at 37 °C.

**Fig. 1.**
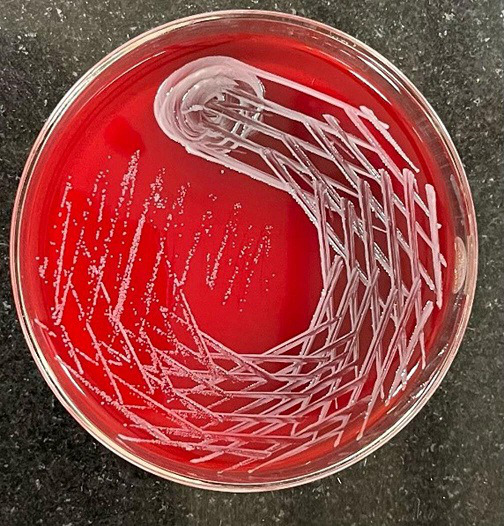
Small, smooth, low convex, transparent non-haemolytic colonies seen on 5 % sheep blood agar.

**Fig. 2.**
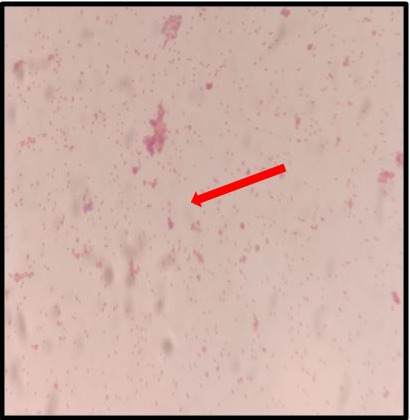
Gram-staining showing Gram-negative coccobacilli under 1000× magnification (red arrow).

Culture growth obtained from all the flagged bottles were identical. The colonies grew to approximately 0.5 mm in size, which were round in shape, smooth, oval, low convex, transparent non-haemolytic after 48 h of aerobic incubation (Fig. 1). Gram-staining of the colonies depicted Gram-negative coccobacilli under 1000× magnification (Fig. 2).

**Fig. 3.**
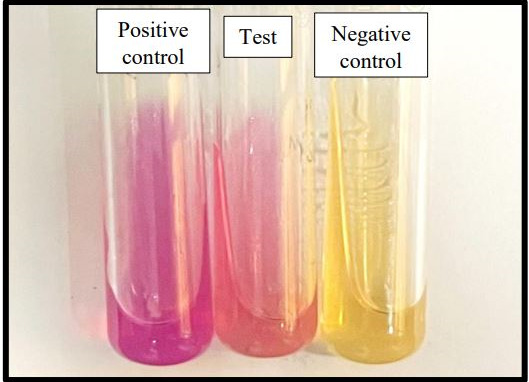
Rapid Urease test positive in 2 mins (centre tube) with negative control (right tube) inoculated with *Escherichia coli* ATCC 25922 and positive control (left tube) inoculated with *Klebsiella pneumoniae* ATCC 70063.

The colonies were processed by VITEK MS (BioMérieux, Inc.) (data: S1.a) and VITEK 2.0 Compact system (BioMérieux, Inc.) (data: S1.b). VITEK MS gave the identification as *Brucella* spp. with 99.9 % of confidence value and VITEK 2.0 Compact system gave identification of *Brucella melitensis* with 99 % probability. The isolated colonies were further confirmed by manual biochemical rapid urease test. Colonies gave positive rapid urease test (Fig. 3). Simultaneously patient’s serum was subjected to Brucella serology by Enzyme Linked Immunosorbent Assay (ELISA), which in turn was found out to be reactive for both anti-Brucella IgM (Optical density: 0.9055) and IgG (Optical density: >4.0) antibody (Calbiotech, Inc Brucella IgM and IgG ELISA Kit) (data: S1.c).

**Fig. 4.**
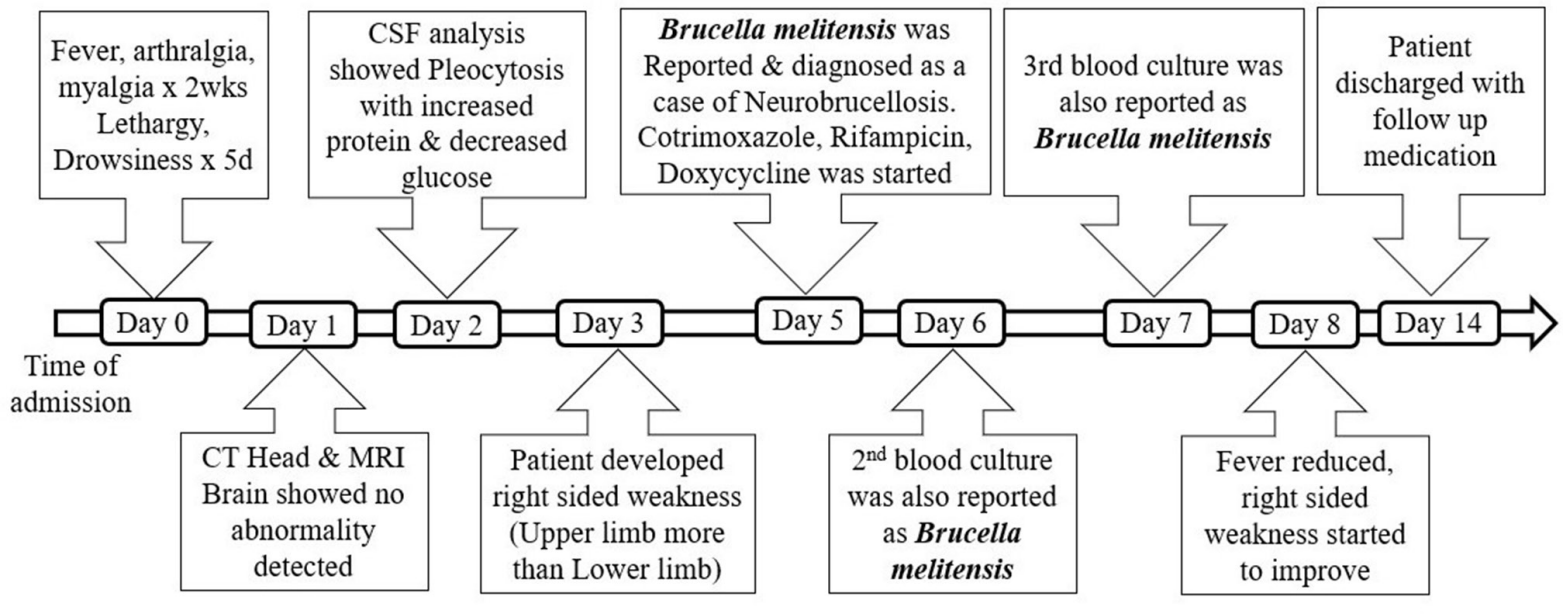
Timeline of events in chronological order.

With above clinical symptoms and microbiological evidence, patient was diagnosed as a case of neurobrucellosis and initiated on the triple drug therapy (Ceftriaxone 2 g once a day, Rifampicin 600 mg once a day and Doxycycline 100 mg twice a day). Clinical and laboratory parameters started to improve on the next day of treatment and right sided weakness started to improve gradually. The patient was discharged 3 days post-initiation of the targeted therapy, with the triple drug therapy (Cotrimoxazole [80/400 mg] once a day for 4 weeks, Rifampicin 600 mg once a day for 3 months and Doxycycline 100 mg once a day for 3 months). Fig. 4 depicts the chronology of events in a sequence.

## Case discussion

In India, brucellosis is regarded as a deceiving infectious disease and the incidence of human brucellosis varies in different studies ranging from 0.8 % in Kashmir, 0.9 % in Delhi, 8.5 % in Gujarat, 11.51 % in Andhra Pradesh, 16.7 % in western Rajasthan, 19.83 % in Maharashtra [6–10]. First case report of neurobrucellosis in western Rajasthan was reported by Kocher DK *et al*. [11] in 2020 from Bikaner. In this study, 12 patients were confirmed as neurobrucellosis by serology alone without any other evidence. Similar studies were reported in Bikaner by Chahar C *et al*. [12] in 2012 and Choudhary MG *et al*. [13] in 2012. After which no studies were reported from western Rajasthan. This can be due to lack of clinical suspicion or non-availability of diagnostic facilities.

Moreover, the symptoms of brucellosis are nebulous and frequently manifest as fever, chills, arthralgia, myalgia, weariness, sweating, foul-smelling perspiration, and anorexia [3]. Hence brucellosis is frequently underdiagnosed and causes multisystem complications, primarily affecting the gastrointestinal tract, hepatobiliary system, and skeletal system. Neurobrucellosis is rare and fatal complication seen in 5–10 % of cases. Meningitis, encephalitis, myelitis, radiculo-neuritis, brain abscess, epidural abscess, peripheral neuropathy and psychosis are all symptoms of neurobrucellosis [14].

The clinical manifestation of central nervous system involvement varies. Meningoencephalitis is the most common kind of nervous system involvement. Basal meningitis can cause severe headache, visual disturbance, and increase intracranial pressure [15]. Guven *et al*. [4] discovered that neurobrucellosis was related with headache, hazy vision, loss of eyesight, hearing loss and confusion. In India, TB is the most common differential diagnosis for brucellosis. In terms of clinical presentation, CSF analysis and neuroimaging, there is a considerable overlap between neurobrucellosis and tuberculosis. In such instances, serological tests like ELISA, standard tube agglutination, rose bengal test, complement fixation, indirect Coombs and immunocapture agglutination (Brucella Capt) plays a vital role in differentiating neurobrucellosis [16]. Because of a lack of consensus on diagnostic criteria, neurobrucellosis is a diagnostic mystery. Diagnosis of neurobrucellosis is established based on several criteria by Kochlar *et al*. [17]: i) Presence of neurological dysfunction that cannot be attributed to other known neurological diseases. ii) Abnormal cerebrospinal fluid (CSF) findings, including lymphocytic pleocytosis (an increase in lymphocytes) and elevated protein levels. iii) Positive CSF culture for Brucella organisms or a positive Brucella IgG agglutination titre detected in both the blood and CSF. iv) A positive response to specific chemotherapy, demonstrated by a decrease in CSF lymphocytic pleocytosis. These diagnostic pillars helped us in giving strong microbiological evidence of the disease.

The prevalence of neurobrucellosis ranges from 1.7–10 % of brucellosis worldwide. *Brucella melitensis* was found in 42 of 2579 blood cultures (1.6 %), with 41 (97.6 %) recognised by Bactec FX Blood culture system within 2 to 6 days and just one culture spotted by blind subculture at the end of the first week. For days 1 to 7, the detection rates by Bactec FX Blood culture system were 0.0, 23.6, 78.9, 86.8, 92.1, 97.6, and 97.6 %, respectively [18], thus implicating the role of blood culture rate in detecting this fatal infection. In a study by Maji S *et al*., in Karnataka, Bangalore, 58.8 % (278/473) cases were positive in serum and/or CSF for brucella by any of the following methods Rose Bengal Plate Test (RBPT), standard tube agglutination test (STAT), indirect enzyme linked immunosorbent assay (iELISA) for IgM and IgG antibodies and polymerase chain reaction (PCR) to detect BCSP31 gene [19].

This case highlights the need of awareness and appropriate serological as well as culture proven investigations for human brucellosis, where in all endemic causes of febrile illnesses are ruled out. In conclusion, it’s important to keep in mind the unusual morbid consequence of human brucellosis known as neurobrucellosis. If detected at an early stage, it is a curable cause of febrile sickness.

## Learning points

- Even in non-endemic areas, neurobrucellosis should be considered if a patient presents as Pyrexia of Unknown Origin (PUO).
- A high level of clinical suspicion even in the absence of radiological indications.
- Early diagnosis and prompt treatment can reduce the morbidity and mortality.

## Supplementary Data

Supplementary material 1
