# Supplementary material for: Unveiling Neurobrucellosis: A Case Report Emphasizing Early Diagnosis for Better Outcomes
Source: Access Microbiol. 2024 Feb 13;6(2):000705.v3. doi: 10.1099/acmi.0.000705.v3 (PMC10928401; doi:10.1099/acmi.0.000705.v3)
Supplement: Supplementary material 1 [file acmi-6-705.v3-s001.pdf]

Supplementary data: S1.a

VITEK® MS Review Detail

Delayed VITEK® MS Review ❯ VITEK® MS Review Detail

Isolate information

Accession ID: 21112022BC94-1 Specimen Type: - Confidence Level:

Number of identifications: 1

List of identifications

	Position	Analysis Date	Organism Name	Pathogenicity	Confidence Value	Confidence Level	Acquisition/Computation message(s)
	I3	11/22/22 12:28 PM	Brucella spp	Highly Pathogenic organism	99.9		

Key: Validate selection Add comment

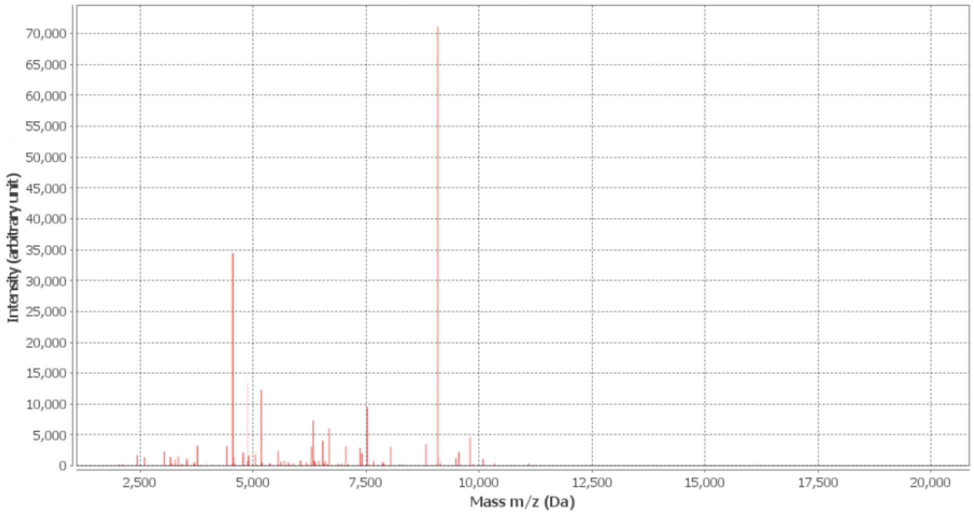

Supplementary data: S1.b

AIIMS JODHPUR

bioMérieux Customer: MICRO | Microbiology Chart Report | Printed November 24, 2022 2:54:13 PM IST

Patient Name: | Patient ID:
Location: | Physician:
Lab ID: BC/94 | Isolate Number: 1

Organism Quantity:
Selected Organism : Brucella melitensis

Source: | **Collected:**

**Comments:**	

**Identification Information**	**Analysis Time:**	7.73 hours	**Status:**	Final
**Selected Organism**	99% Probability	**Brucella melitensis**		
	**Bionumber:**	0000001300001000		
**ID Analysis Messages**				

Biochemical Details

2	APPA	-	3	ADO	-	4	PyrA	-	5	lARL	-	7	dCEL	-	9	BGAL	-
10	H2S	-	11	BNAG	-	12	AGLTp	-	13	dGLU	-	14	GGT	-	15	OFF	-
17	BGLU	-	18	dMAL	-	19	dMAN	-	20	dMNE	-	21	BXYL	-	22	BAlap	-
23	ProA	+	26	LIP	-	27	PLE	-	29	TyrA	+	31	URE	+	32	dSOR	-
33	SAC	-	34	dTAG	-	35	dTRE	-	36	CIT	-	37	MNT	-	39	5KG	-
40	lLATk	-	41	AGLU	-	42	SUCT	-	43	NAGA	-	44	AGAL	-	45	PHOS	-
46	GlyA	+	47	ODC	-	48	LDC	-	53	lHISa	-	56	CMT	-	57	BGUR	-
58	O129R	-	59	GGAA	-	61	lMLTa	-	62	ELLM	-	64	lLATa	-			

Supplementary data: S1.c

ELISA CHART

Name of test: Ig M Brucella

Kit Manufacturer: CALBIOTECH

Date of Test: 23/11/22

Test performed by: Dr. S. Haripriya.

ROW	1	2	3	4	5	6	7	8	9	10	11	12
A	Blank	1:32										
	0.0062	0.0014										
B	NC	1:64										
	0.0472	0.0212										
C	PC											
	1.8957											
D	Cali.											
	1.5874											
E	1:2											
	0.9055											
F	1:4											
	0.3831											
G	1:8											
	0.1401											
H	1:16											
	0.0018											

Cutt off = 1.5874 × 0.395

= 0.627

LisaScan EM

Serial No :180222

S/w Ver :2017.00.02

Zero Initialization :YES

Date:23/11/22 Time:11:11

Plate I.D.:101

FILTERS :1 450 2 630

SMPL ID ABSORBANCE

01 0.0062	02 0.0472	03 1.8957
04 1.5874	05 0.9055	06 0.3831
07 0.1401	08 0.0018	09 0.0014
10 0.0212	11 0.0017	12 0.0018
13 0.0012	14 0.0016	15 0.0011
16 0.0006	17 0.0008	18 0.0023
19 0.0005	20 0.0016	21 0.0009
22 0.0030	23 0.0011	24 0.0005
[illegible]	26 [illegible]	27 0.0020

CALCULATIONS OF CUT OFF-

SIGN

S. Har.

Supplementary data: S1.c

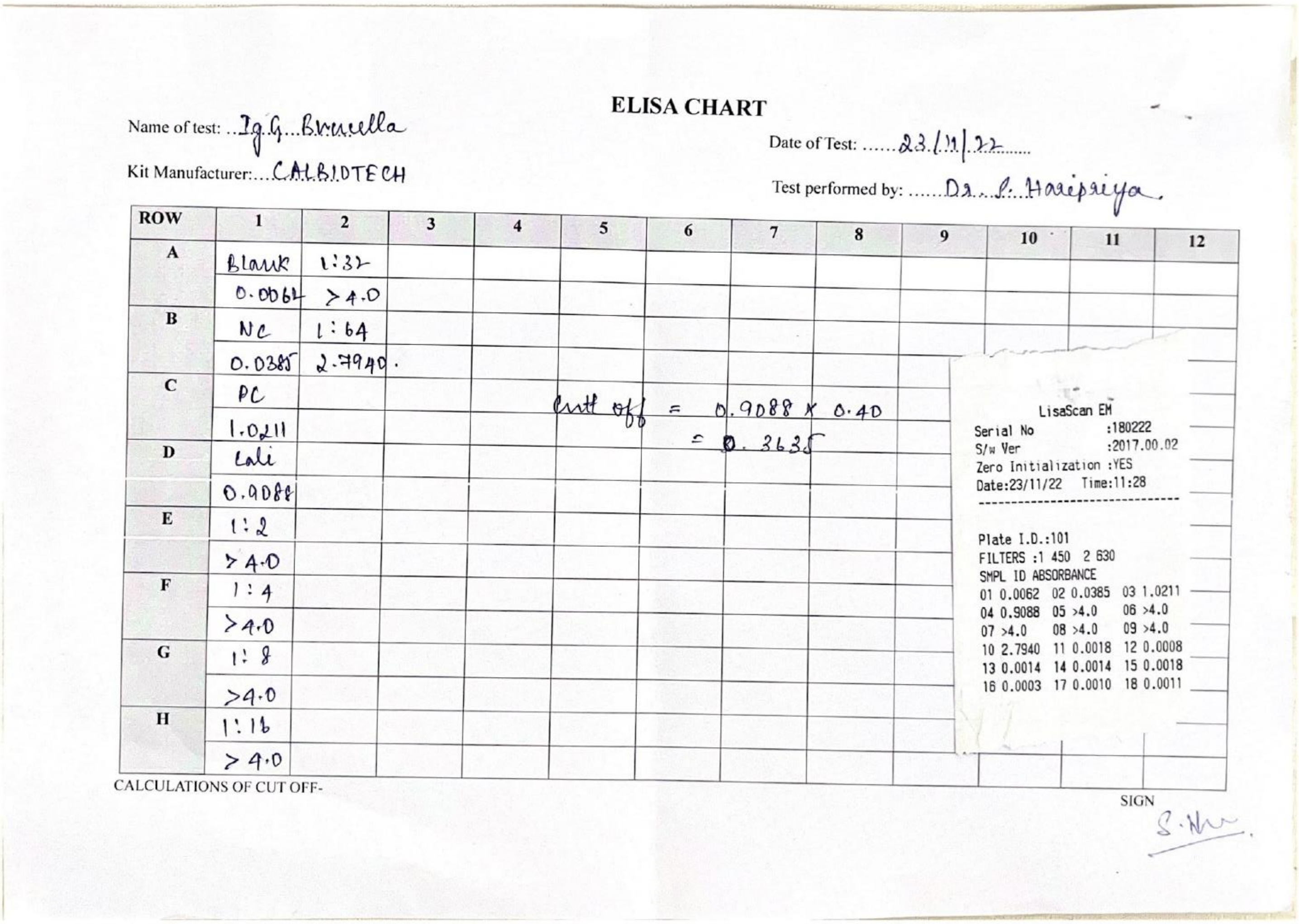

ELISA CHART

Name of test: Ig.G. Brucella

Kit Manufacturer: CALBIOTECH

Date of Test: 23/11/22

Test performed by: Dr. S. Haripriya.

ROW	1	2	3	4	5	6	7	8	9	10	11	12
A	Blank	1:32										
	0.0062	>4.0										
B	NC	1:64										
	0.0385	2.7940.										
C	PC											
	1.0211											
D	Cali											
	0.9088											
E	1:2											
	>4.0											
F	1:4											
	>4.0											
G	1:8											
	>4.0											
H	1:16											
	>4.0											

Cutt off = 0.9088 x 0.40
= 0.3635

LisaScan EM
Serial No :180222
S/w Ver :2017.00.02
Zero Initialization :YES
Date:23/11/22 Time:11:28

Plate I.D.:101
FILTERS :1 450 2 630
SMPL ID ABSORBANCE
01 0.0062 02 0.0385 03 1.0211
04 0.9088 05 >4.0 06 >4.0
07 >4.0 08 >4.0 09 >4.0
10 2.7940 11 0.0018 12 0.0008
13 0.0014 14 0.0014 15 0.0018
16 0.0003 17 0.0010 18 0.0011

CALCULATIONS OF CUT OFF-

SIGN

S. Hr.
